# Effect of skipping breakfast on cardiovascular risk factors: a grade-assessed systematic review and meta-analysis of randomized controlled trials and prospective cohort studies

**DOI:** 10.3389/fendo.2023.1256899

**Published:** 2023-11-28

**Authors:** Junhui Yu, Jiayue Xia, Dengfeng Xu, Yuanyuan Wang, Shiyu Yin, Yifei Lu, Hui Xia, Shaokang Wang, Guiju Sun

**Affiliations:** ^1^ Key Laboratory of Environmental Medicine and Engineering of Ministry of Education, School of Public Health, Southeast University, Nanjing, China; ^2^ Department of Nutrition and Food Hygiene, School of Public Health, Southeast University, Nanjing, China

**Keywords:** anthropometrics, blood pressure, cardiovascular risk factors, glycemic control, lipid profiles, meta-analysis, skipping breakfast, systematic review

## Abstract

Skipping breakfast is one of the most prevalent irregular eating habits. Several pieces of evidence have reported the association between breakfast omission and a higher risk of cardiovascular diseases. Numerous publications have focused on the impact of skipping breakfast on various cardiovascular risk factors. Therefore, the current systematic review and meta-analysis aimed to assess this impact, especially with regard to anthropometric measurements, serum lipid profiles, blood pressure, and glycemic control indicators. A comprehensive search was performed in PubMed, Web of Science, Embase, Scopus, and the Cochrane Central Register of Controlled Trials up to 1 April 2023. A total of 11 eligible trials were identified to evaluate the combined effects of skipping breakfast. Final integrated results demonstrated that breakfast omission significantly decreased the body weight (mean difference = −0.66, 95% CI: −1.09 to −0.24, *p* = 0.002, *I*^2 =^ 0.0) and increased the level of serum low-density lipoprotein cholesterol (LDL-C) (mean difference = 9.89, 95% CI: 5.14 to 14.63, *p* = 0.000, *I*^2 =^ 17.3). Subgroup analysis also revealed potential factors that may affect the outcomes, for example, the physiological condition of participants, duration, gender, and type of breakfast. In conclusion, skipping breakfast may reduce body weight while increasing the level of serum LDL-C at the same time. In view of the limited trials, further studies are needed to expound the role of breakfast omission in cardiovascular diseases.

## 1 Introduction

Breakfast is generally considered as the first meal before ten o’clock in the morning. Skipping breakfast is defined as the omission of any food except water (1). For a long time, it has been widely accepted that breakfast holds significant importance as the first meal of the day, playing a crucial role in health behaviors, although its exact mechanism remains unclear (2). Human diets have changed drastically over the past few decades due to the transformation of lifestyles. According to a nationally representative survey in Canada, 11% of adults never took breakfast (3). Similarly, data from National Health and Nutrition Examination Survey (NHANES) 1999–2014 reported that more than one in five US adults skipped breakfast. The consumption of breakfast had a dismal situation (4–6).

According to the data from the American Heart Association (AHA), more than 26 million adults in the United States suffer from cardiovascular diseases (CVDs), excluding hypertension (7). In 2018, cardiovascular disease accounted for approximately one-third of all U.S. deaths, becoming a huge burden on human health and society (8, 9). These situations are not just confined to the US as cardiovascular diseases have long been a leading cause of death globally (10). Predictions for 2025 and 2060 suggest a substantial increase in the prevalence of cardiovascular diseases (11). It was reported that a potential risk factor for cardiovascular disease is irregular or inconsistent diet, for example, skipping breakfast. Results from a British birth cohort indicated that irregular diet positively associated with cardiometabolic risk (12). A national representative prospective cohort study demonstrated a significant increase in the risk of mortality from cardiovascular disease associated with skipping breakfast (13). Several studies have reached the same conclusion (14–17). However, skipping breakfast was also considered as an intermittent fasting strategy that may benefit the development of cardiovascular disease in specific situations. When considering the chrononutrition nutritional physiology, taking breakfast was related to the endocrine pancreatic clock and insulin secretion, which ultimately enhance the metabolic pathways related to cardiovascular diseases (18). In addition, many disease states including obesity, raised blood cholesterol, and raised blood pressure are regarded as risk factors for cardiovascular diseases. Nevertheless, few studies have elaborated on the effect of skipping breakfast on them, making it necessary to conduct this systematic review and meta-analysis to clarify it.

## 2 Method

### 2.1 Search strategy and eligibility criteria

A comprehensive systematic search was conducted in PubMed, Web of Science, Embase, Scopus, and the Cochrane Central Register of Controlled Trials from database inception to 1 April 2023. Preferred Reporting Items for Systematic Reviews and Meta-analysis (PRISMA) Guideline was followed to evaluate the effect of skipping breakfast on cardiovascular risk factors (19). The method of subject words combined with free words was adopted to perform a retrieval scheme. These search terms were used to identify the relevant literature: (“breakfast” OR “morning meal” OR “skip breakfast” OR “skipping breakfast” OR “breakfast omission” OR “omit breakfast” OR “fasting”) AND (“cardiovascular disease” OR “cardiovascular disease risk factors” OR “weight” OR “body mass index (BMI)” OR “waist circumference” OR “waist-to-hip ratio (waist:hip ratio)” OR “lipid profiles” OR “high-density lipoprotein cholesterol (HDL-C)” OR “low-density lipoprotein cholesterol (LDL-C)” OR “total cholesterol (TC)” OR “total triglyceride (TG)” OR “diastolic blood pressure (DBP)” OR “systolic blood pressure (SBP)” OR “glucose” OR “blood glucose” OR “blood sugar” OR “fasting blood sugar (FBS)” OR “fasting plasma glucose (FPG)” OR “fasting blood glucose (FBG)” OR “glycated hemoglobin (HbA1c)” OR “insulin resistance index (HOMA-IR)”). Overall, these indicators can be divided into four classes: anthropometrics, serum lipid profiles, blood pressure, and glycemic control indicator. No limitations were set for the limited relevant trials. Literature would be included if they fulfill the following inclusion criteria (1): Randomized controlled trials or prospective cohort studies (2). Trials aiming to probe the effect of skipping breakfast on the factors mentioned above (3). Participants of the intervention group must finish breakfast before 10:00, without limitations on the type or the features of breakfast (4). Participants of the control group consume nothing but water. The exclusion criteria were as follows (1): Case reports, observational studies, and retrospective studies (2). Participants with special pathological conditions (for example, fasting because of surgery) (3). Trials including pharmacological or dietary supplement interventions. Literature search was performed by two reviewers (J.Y. and J.X.) independently. To ensure the rigor of the retrieval strategy, we also hand-searched and checked the reference lists of included articles and relevant reviews. Any disagreement in this progress has been discussed and resolved.

### 2.2 Data extraction and quality assessment

Two authors (J.Y. and Y.L.) collected the characteristics of 11 included trials independently including (a) name of the first author, year of publication, and study region; (b) sample size, number of male and female participants, mean age of participants, study design, and duration; and (c) health status of participants, intervention details, and outcomes of interest. A third reviewer (D.X. or Y.W.) checked the extracted data in case of any errors. Cochrane Risk of Bias 2.0 tool (RoB2) was conducted to assess the risk of bias in RCTs including four domains: bias due to the randomization process, deviations from intended interventions, missing outcome data, and selection of the reported results (20). Meanwhile, GRADE was adopted to evaluate the evidence level of RCTs while the Newcastle–Ottawa Scale (NOS) was used for cohort studies. Quality was rated as high, medium, low, and very low according to the risk of bias, inconsistency, indirectness, imprecision, and publication bias (21). Publication bias was assessed via Egger’s tests and funnel plots, and trim and fill methods for any asymmetry if it exists (22). If there existed multiple intervention groups in the trials, the breakfast and no-breakfast groups were combined to explore the effect of skipping breakfast on the indicators mentioned above.

### 2.3 Data integration and quantitative analysis

The data of interest were presented in the form of mean and standard error or 95% confidence intervals (CIs). For data only in graphs or figures, we contacted the original authors if possible. For parallel-controlled trials, mean difference (MD) was calculated by the change from baseline in both control and intervention groups. Moreover, the value of the last endpoint was selected to analyze if there was more than one endpoint while the final level of cardiovascular risk factors was extracted to compute the MD in crossover trials. The following formula was used if the net change of SD is not provided:


$$\begin{array}{l} \text{SDnet change}\\ \;=\sqrt{\text{SD}{\left(\text{baseline}\right)}^{2}+\text{SD}{\left(\text{baseline}\right)}^{2}-2\text{R}\times \text{SD}(\text{baseline})\times \text{SD}(\text{endpoint})} \end{array}$$

based on a correlation coefficient *R* = 0.5 (23). Units of the following indicators were harmonized as mg/dL including serum lipid profiles and fasting glucose. *I*^2^ and chi-squared statistics were adopted as a measure of heterogeneity, only *I*^2^< 50% and *p*-value > 0.05 were considered as no statistical heterogeneity. Based on the heterogeneity test, either a random‐ or fixed‐effect model was chosen for pooled results. The method of leave-one-out sensitivity analysis was used to identify the heterogeneity, and so was subgroup analysis. Leave-one-out sensitivity analysis performs multiple meta-analyses by excluding one study at each analysis to determine the impact and effect of individual studies or data in the context of overall data (24). All of the statistical analyses above were conducted via Stata version 15 software (STATA Corp, College Station, TX, USA).

## 3 Results

### 3.1 Literature search

**Figure 1:**
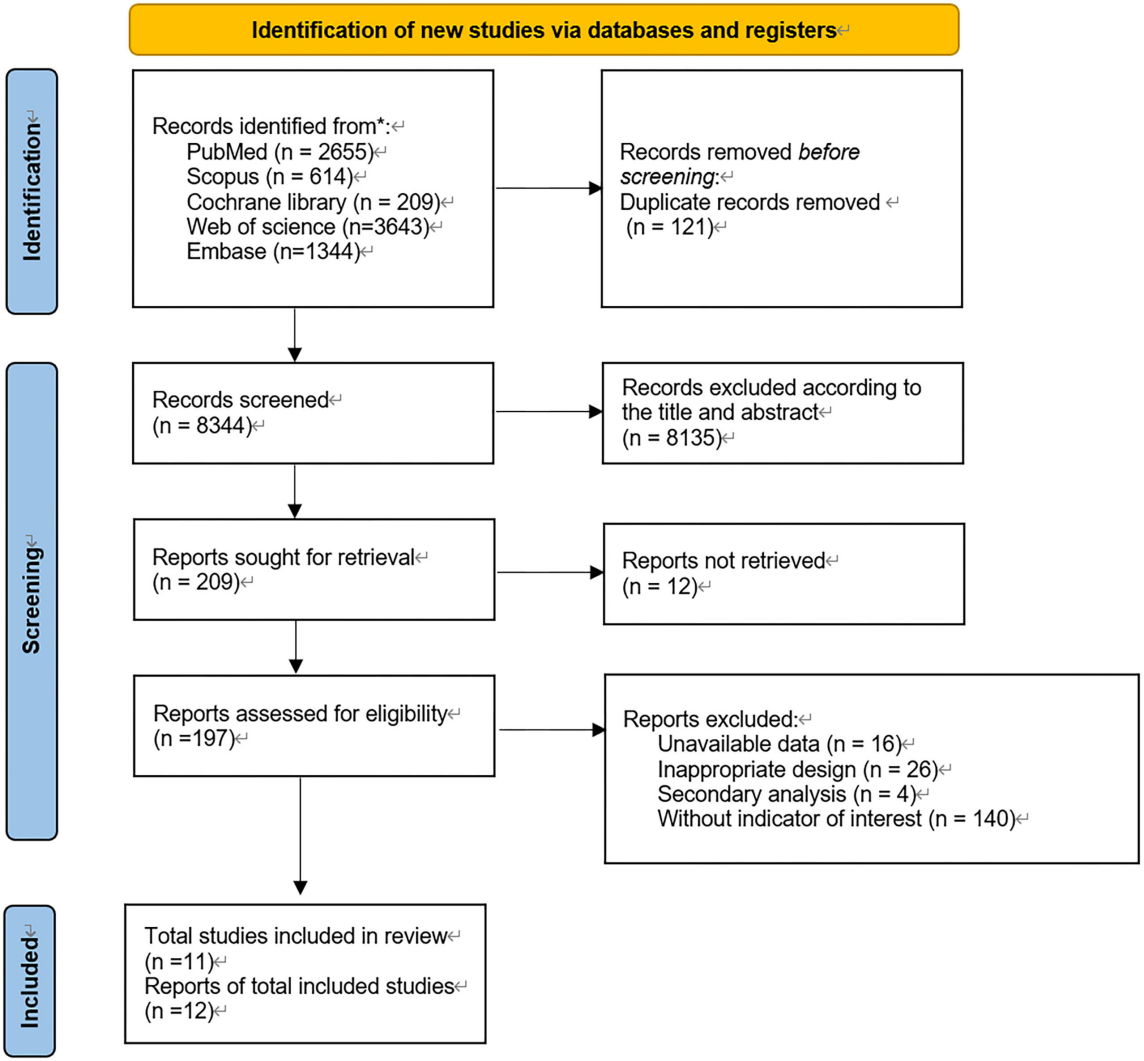
Flowchart.

The detailed screening methodology is presented in **Figure 1**. A total of 8,465 pieces of literature were identified initially by the search strategy. Duplicate records were removed, resulting in 8,344 remaining articles. After browsing the title and abstract, 209 articles were selected for further intensive reading. Final screening led to 11 pieces of literature included in the current systematic review and meta-analysis.

### 3.2 Characteristics of the trials

The 11 studies evaluating the effect of skipping breakfast on cardiovascular risk factors were included in this systematic review, namely, 9 RCTs, 1 randomized crossover trial, and 1 cohort study (25–35). The publication year of trials ranged from 1992 to 2019. In total, 1,118 participants aged from 11 to 55 were allocated to the breakfast group or breakfast skipping group. Most of the participants were healthy individuals, and only 4 trials were conducted among patients with overweight, obesity, or metabolic syndrome. Study duration ranged from 2 days to 10 years, and the majority of the trials (*n* = 10) had an intervention duration of more than 4 weeks. The detailed summary is shown in **Table 1**.

**Table 1:** Characteristics of included studies.

Author, year	Country	Size, *n*	Male/female, *n*	Mean age (years)	Ault or minor	Study design	Duration	Health status of participants	Intervention group	Control group	Outcome
Geliebter 2014	USA	36	18/18	33.4 ± 6.5	Adult	Randomized, controlled parallel-arm	1 month	Healthy	Consumption of oat porridge, frosted cornflakes within 15 min at 8:30	Breakfast skipping	Weight; waist; waist:hip ratio; SBP; DBP; HDL-C; LDL-C; TC; TG; fasting glucose; HOMA-IR
Neumann 2016	USA	24	0/24	24.1 ± 2	Adult	Randomized, controlled	8 days	Healthy	Consumption of protein-based breakfast or carbohydrate-based breakfast	Breakfast skipping	Weight; BMI
Goff 2019	UK	665	329/336	22.7	Minor and adult	Cohort	10 years	Healthy	Consumption of regular breakfast	Breakfast skipping	BMI; waist:hip ratio; SBP; HDL-C; TC; HbA1c
Kobayashi 2013	Japan	8	8/0	25.3 ± 1.2	Adult	Randomized, crossover	2 days	Healthy	Consumption of meals at predetermined time: breakfast (8:00), lunch (12:00), and supper (19:00)	Breakfast skipping, lunch (12:00), and supper (19:00)	Fasting glucose
Zhang 2017	USA	178	48/130	52.2	Adult	Randomized, controlled	1 year	Metabolic syndrome	Consumption of regular breakfast	Breakfast skipping	Weight; BMI; waist; SBP; DBP; HDL-C; LDL-C; TC; TG; HbA1c; fasting glucose; HOMA-IR
Schlundt 1992	USA	38	0/38	18–55	Adult	Randomized	3 months	Obesity	Consumption of breakfast (1,672 kJ), lunch (1,254 kJ), and supper (2,090 kJ)	Consumption of lunch (1,254 kJ) and supper (2,090 kJ) without breakfast	Weight
Leidy 2015	USA	54	22/32	19 ± 1	Adult	Randomized, controlled	3 months	Overweight, obesity	Consumption of 1,464 kJ breakfast (13 g protein) or a high-protein breakfast (35 g protein)	Breakfast skipping	Weight, BMI
LeCheminant 2016	USA	49	0/49	23.7 ± 6.8	Adult	Randomized, controlled	1 month	Healthy	Consumption of at least 15% of daily energy requirement before 8:30	Without consuming any energy until 11:30	Weight, BMI
Chowdhury 2016	UK	23	8/15	44 ± 10	Adult	Randomized, controlled	6 weeks	Obesity	Consumption of daily breakfast (at least 700 kcal)	Extended fasting (0 kcal until 12:00)	Weight; BMI; waist; waist:hip ratio; HDL-C; LDL-C; TC; TG; fasting glucose; HOMA-IR
Betts 2014	UK	33	12/21	36 ± 11	Adult	Randomized, controlled	6 weeks	Healthy	Consumption of daily breakfast (at least 700 kcal)	Extended fasting (0 kcal until 12:00)	Weight; BMI; waist; waist:hip ratio; SBP; DBP; HDL-C; LDL-C; TC; TG; fasting glucose; HOMA-IR
Farshchi 2005	UK	10	0/10	25.5 ± 5.7	Adult	Randomized, crossover	1 month	Healthy	Consumption breakfast cereal with 2% fat milk before 8:00	Breakfast skipping	Weight; BMI; waist; waist:hip ratio; HDL-C; LDL-C; TC; TG; fasting glucose

### 3.3 Risk of bias and grading of the evidence

Full details of the risk of bias and grading of the evidence are shown in **Supplementary Appendix.** Among 11 trials included in this systematic review, five RCTs were rated for high risk of the lack of blinding in the randomization process, deviations from the intended interventions, and missing outcome data. Meanwhile, due to the inconsistency and imprecision of the outcome variables, most of the evidence was graded as low or moderate. Publication bias was also presented in **Supplementary Appendix.**

### 3.4 Effect of skipping breakfast on cardiovascular risk factors

#### 3.4.1 Anthropometrics

**Figure 2:**
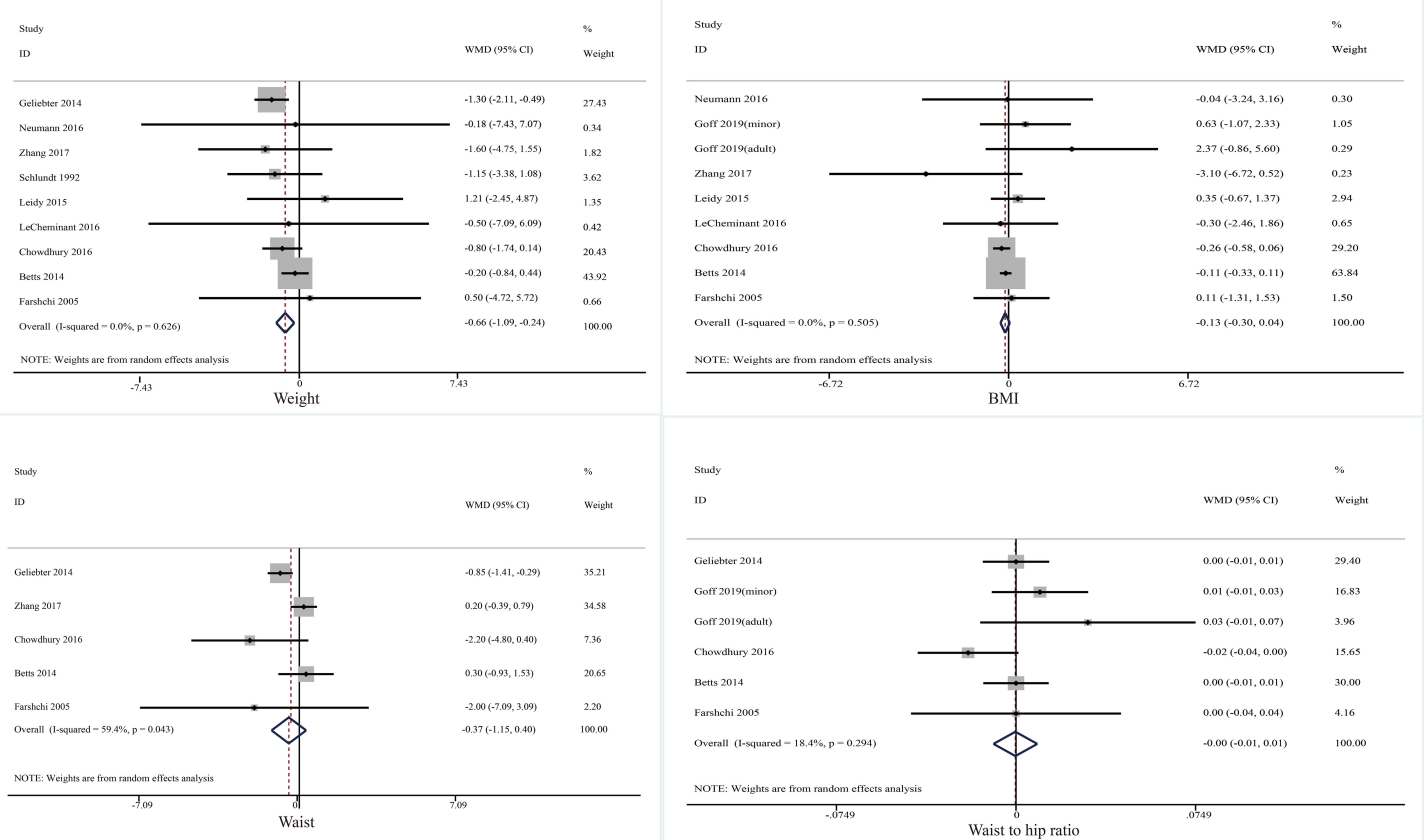
Effect of skipping breakfast on anthropometrics.

Ten trials investigated the effect of skipping breakfast on anthropometrics (**Figure 2**). Compared to taking breakfast, skipping breakfast could reduce weight significantly (mean difference = −0.66, 95% CI: −1.09 to −0.24, *p* = 0.002, *I*^2 =^ 0.0). No significant changes were observed in other indicators: BMI: mean difference = −0.13, 95% CI: −0.30 to 0.04, *p* = 0.144, *I*^2 =^ 0.0; Waist circumference: mean difference = −0.37, 95% CI: −1.15 to 0.40, *p* = 0.344, *I*^2 =^ 59.4; waist-to-hip ratio: mean difference = 0.00, 95% CI: −0.01 to 0.01, *p* = 0.344, *I*^2 =^ 59.4.

#### 3.4.2 Serum lipid profiles

**Figure 3:**
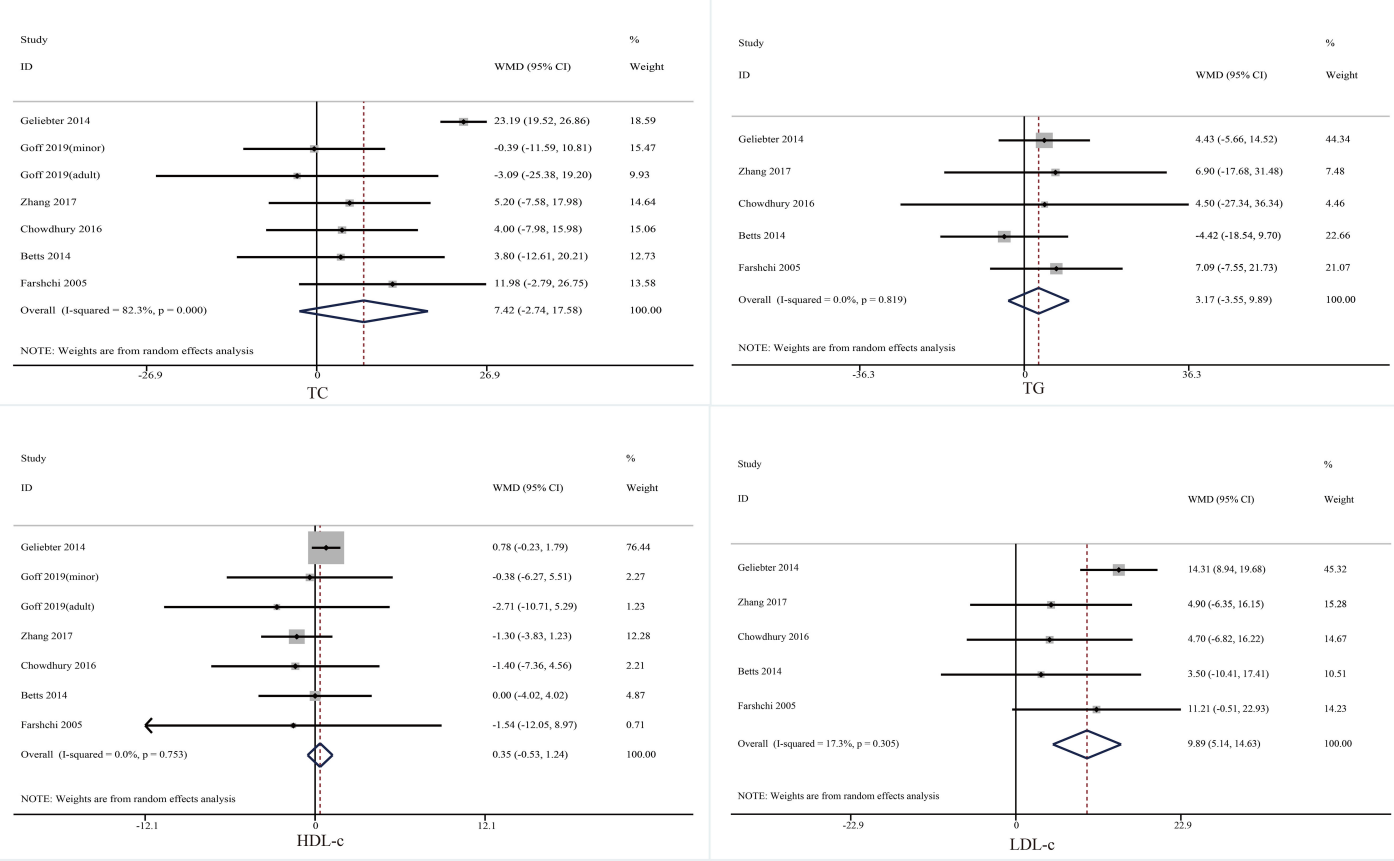
Effect of skipping breakfast on serum lipid profiles.

Six trials probed the effect of skipping breakfast on serum lipid profiles (**Figure 3**). The merged data indicated that skipping breakfast did cause a significant increase in serum LDL-C (mean difference = 9.89, 95% CI: 5.14 to 14.63, *p* = 0.000, *I*^2 =^ 17.3). No statistical significance was observed in the remaining indicators: serum TC: mean difference = 7.42, 95% CI: −2.74 to 17.58, *p* = 0.152, *I*^2 =^ 41.0; serum TG: mean difference = 3.17, 95% CI: −3.55 to 9.89, *p* = 0.355, *I*^2 =^ 0.0; serum HDL-C: mean difference = 0.35, 95% CI: −0.53 to 1.24, *p* = 0.435, *I*^2 =^ 0.0.

##### 3.4.2.1 Blood pressure

**Figure 4:**
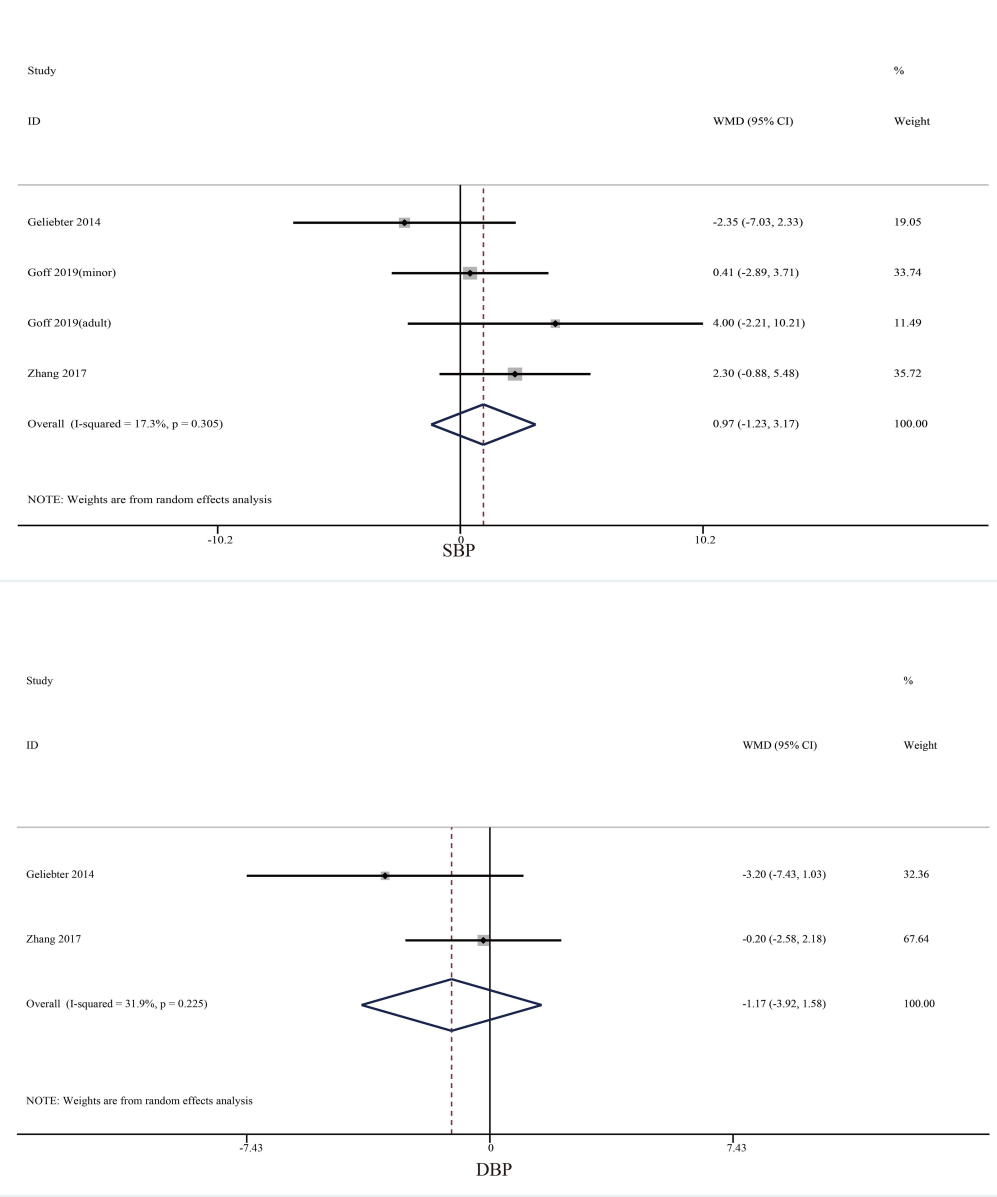
Effect of skipping breakfast on blood pressure.

Four studies focused on the effect of skipping breakfast on blood pressure (**Figure 4**). No significant changes were reported either among SBP (mean difference = 0.97, 95% CI: −1.23 to 3.17, *p* = 0.386, *I*^2 =^ 17.3) or DBP (mean difference = −1.17, 95% CI: −3.92 to 1.58, *p* = 0.404, *I*^2 =^ 31.9).

##### 3.4.2.2 Glycemic control indicators

**Figure 5:**
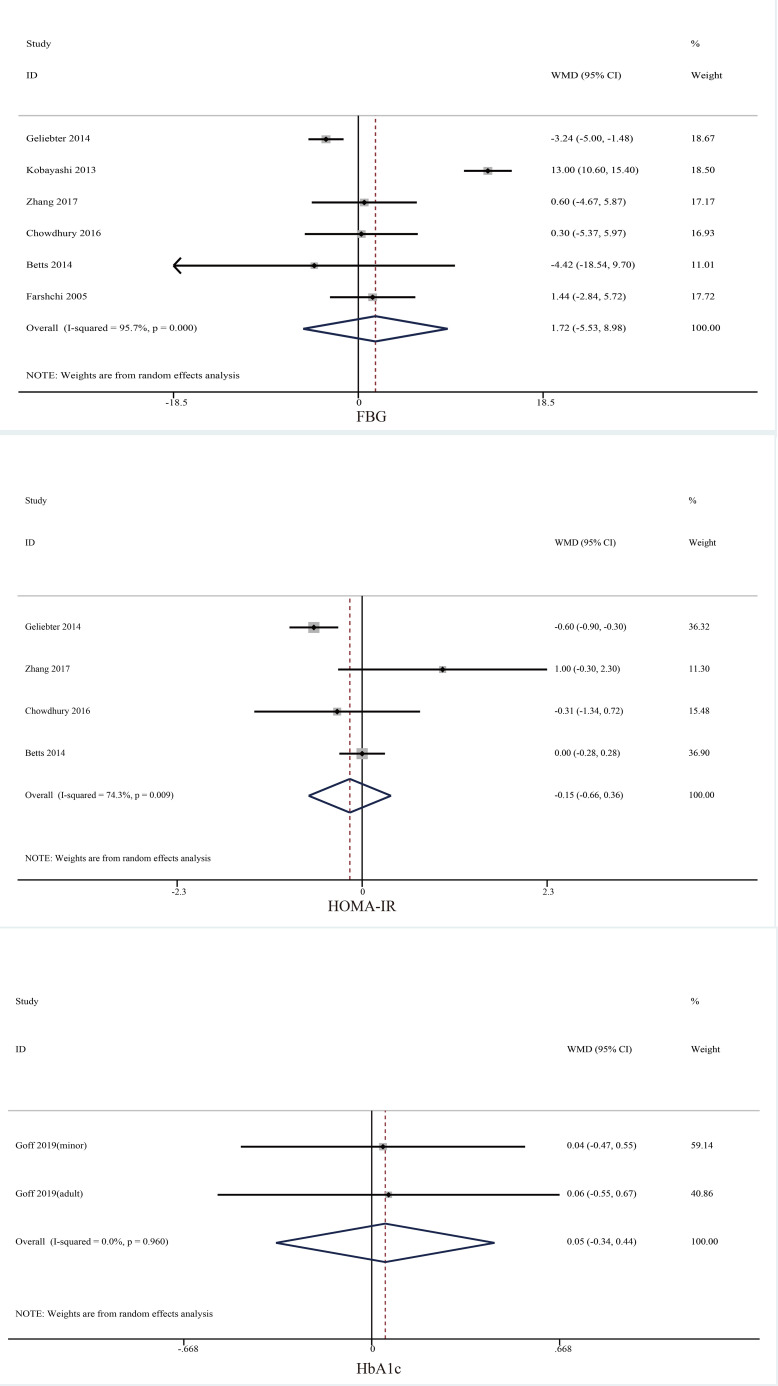
Effect of skipping breakfast on glycemic control indicators.

Seven trials reported the effect of skipping breakfast on glycemic control indicators (**Figure 5**). However, no significant changes were found in FBG (mean difference = 0.00, 95% CI: −0.05 to 0.05, *p* = 0.642, *I*^2 =^ 97.2), HbA1c (mean difference = 0.05, 95% CI: −0.34 to 0.44, *p* = 0.808, *I*^2 =^ 96.0), and HOMA-IR (mean difference = −0.15, 95% CI: −0.66 to 0.36, *p* = 0.558, *I*^2 =^ 74.3).

##### 3.4.2.3 Subgroup analysis

Subgroup analysis was performed based on the physiological condition of the participants, duration of the trials, gender, intervention of the control groups, and the design of the studies. Very little literatures focusing on DBP and HbA1c led to the failure of their subgroup analysis. A series of subgroup analyses elucidated that participants with overweight/obesity or metabolic syndrome (mean difference = −0.80, 95% CI: −1.62 to −0.24, *p* = 0.054, *I*^2 =^ 0.0) tend to lose more weight compared with healthy individuals (mean difference = −0.64, 95% CI: −1.26 to −0.03, *p* = 0.410, *I*^2 =^ 11.9). Moreover, the level of serum LDL-C among healthy people (mean difference = 12.50, 95% CI: 7.63 to 17.73, *p* = 0.000, *I*^2 =^ 4.2) was higher than that of other participants (mean difference = 4.80, 95% CI: −3.24 to 12.85, *p* = 0.242, *I*^2 =^ 0.0). Outcome measures, especially weight, waist circumference, serum LDL-C, and TC, were affected by the treatment duration (weight: ≤4 weeks, mean difference = −1.23, 95% CI: −2.02 to −0.44, *p* = 0.002, *I*^2 =^ 0.0; >4 weeks, mean difference = −0.43, 95% CI: −0.93 to 0.07, *p* = 0.094, *I*^2 =^ 0.0; waist circumference: ≤4 weeks, mean difference = −0.86, 95% CI: −1.42 to 0.37, *p* = 0.258, *I*^2 =^ 34.3; >4 weeks, mean difference = 0.00, 95% CI: −0.87 to 0.87, *p* = 0.995, *I*^2 =^ 37.6; serum LDL-C: ≤4 weeks, mean difference = 13.77, 95% CI: 8.89 to 18.66, *p* = 0.000, *I*^2 =^ 0.0; >4 weeks, mean difference = 4.48, 95% CI: −2.49 to 11.44, *p* = 0.208, *I*^2 =^ 0.0; serum TC: ≤4 weeks, mean difference = 19.96, 95% CI: 10.01 to 29.91, *p* = 0.000, *I*^2 =^ 52.0; >4 weeks, mean difference = 2.41, 95% CI: −3.70 to 8.52, *p* = 0.440, *I*^2 =^ 0.0). Gender and type of breakfast also had their role in subgroup analysis. More details of subgroup analysis are presented in **Supplementary Appendix.**

## 4 Discussion

This meta-analysis, involving 11 trials, summarized the effects of skipping breakfast on cardiovascular risk factors, including anthropometrics, serum lipid profiles, blood pressure, and glycemic control indicators. It conclusively demonstrated that skipping breakfast can lead to weight reduction and elevated levels of serum LDL-C. However, there was no conclusive evidence to certify the relationship between breakfast skipping and other cardiovascular risk factors. Breakfast skippers tend to lose 0.66 kg weight than those who took breakfast. Compared to having breakfast, breakfast skipping reduced the level of serum LDL-C by 9.89 mg/dL. The overall results of this meta-analysis were similar to previous articles (17, 36). Subgroup analysis based on literature characteristics provided more findings.

Several meta-analyses, focusing on RCTs and cohort studies, have consistently reached the conclusion that skipping breakfast may lead to weight reduction in both adults and minors (37, 38). Moreover, observational studies have reported that breakfast acts as a protective factor against overweight or obesity, and this effect is particularly pronounced among male participants (39). The primary mechanism behind this effect is believed to involve hormones that regulate appetite and energy balance, specifically leptin and ghrelin (40). Chowdhury and Betts et al. (26, 27) reported that skipping breakfast may decrease the level of leptin while another trial only observed a decreasing tendency of leptin in the fasting group than those who took breakfast. Fasting, especially short-term fasting, was reported to have the capacity to reduce the level of leptin (41, 42). Notably, a low level of leptin was associated with weight loss, which may partly explain the effect of skipping breakfast in reducing body mass (43, 44). Similar to leptin, ghrelin also has its role in controlling weight. Both leptin and ghrelin engage in energy balance through the hypothalamus and finally contribute to weight loss and leptin has a stronger effect (45–47). Furthermore, leptin is involved in the regulation of signal pathways related to obesity, for example, inflammation and endoplasmic reticulum stress (48–50). Ghrelin also joins the feeding behavior by regulating the responsiveness over satiety signals (51). Although breakfast omission may decrease satiety and result in a daily reduction in energy intake, this effect is prone to be transient. The debate regarding whether energy intake or energy expenditure takes precedence in weight regulation when skipping breakfast remains controversial (52, 53). It should be noted that hormones mentioned above refer to serum leptin and serum ghrelin rather than circulating leptin and ghrelin.

Interestingly, both the duration and the health states of participants revealed different effects on the final pooled results. A recent meta-analysis specifically considered trials lasting more than 4 weeks for data analysis, as there might be a positive correlation between significant weight loss in 1 month and greater weight loss over the long term (17, 54, 55). We acknowledged and considered diverse perspectives on this matter. Our subgroup analysis suggested that skipping breakfast within 1 month may cause more weight loss. Most of the literature focusing on breakfast or skipping breakfast has explored the short-term effects, typically with a duration of less than 4 weeks (56–59). The distinction between short- and long-term effects is not clearly defined. As discussed above, skipping breakfast can lead to short-term weight loss due to hormonal effects, and the majority of trials were conducted within 24 hours, 3 days, or 1 week. It is plausible that short-term omission of breakfast has a stronger impact on weight loss.

Reeves et al. (60) reported that healthy and overweight participants tend to differ in their patterns of energy intake consumption. The breakfast group showed a higher energy intake compared to the no-breakfast group. Notably, participants in the breakfast-skipping group consumed more energy in the afternoon than those who had breakfast, suggesting variable effects among individuals with different BMIs (normal weight, overweight, and obesity). Upon dividing all participants into two groups, we observed that individuals with a healthy weight lost less weight than those classified as having an unhealthy weight. The study demonstrated that neither prolonged morning fasting nor increased afternoon appetite was associated with compensatory intake during ad libitum lunch in obese adults. Interestingly, it appeared that obese individuals were suppressed by ghrelin, regardless of the duration of the overnight fast, which was contrary to common expectations (56). Breakfast skipping did not seem to significantly affect appetite, implying no extra energy intake due to the absence of breakfast. Consequently, the daily energy intake and meal frequency of the breakfast-skipping group were less than those of the breakfast group. Paoli et al. (61) categorized breakfast omission as a form of fasting, potentially explaining the results mentioned earlier. The most recent review indicated that fasting had different effects on weight loss among normal-weight, overweight, and obese individuals, although those of normal weight lost a higher percentage of weight. This may be attributable to the large weight base of the obese since this is a simple mathematical phenomenon of absolute value and percentage. We reached this possible reason with trepidation because of few relevant literature. Whether male or female participants have a better weight loss effect is still unclear because of inconsistent conclusions (62–64). We tried to stratify the analysis by gender but failed, and most of the studies were conducted in both male and female participants. Gender difference is open to debate.

LDL-C is widely considered a significant risk and predictive factor for cardiovascular diseases (65, 66). The pooled results demonstrated that skipping breakfast could lead to elevated levels of serum LDL-C, aligning with findings from several previous studies (28, 67, 68). Physical inactivity and an inadequate diet have been associated with increased components of metabolic syndrome. Unhealthy practices like skipping breakfast are linked to a rise in serum LDL-C levels (69). Both short- and long-term studies have shown that omitting breakfast is associated with increased serum LDL-C levels (28, 70). Moreover, when considering duration (4 weeks) as a stratification factor, the results suggested a more pronounced tendency of elevated serum LDL-C levels when breakfast was omitted within 4 weeks compared to durations exceeding 4 weeks. Although subgroup analysis for durations exceeding 4 weeks showed no significance, observational studies reported opposing results, which warrant further investigation (25, 71).

Several publications have explored the relationship between skipping breakfast and serum TC, primarily focusing on adolescents (72–74). However, only one study from Korea reported a significantly higher level of serum TC in adults who skipped breakfast (75). It appears that the short-term effect of breakfast on serum TC may not be strongly supported due to a lack of sufficient research literature. Shifting the focus to blood pressure, cross-sectional observational studies have indicated elevated blood pressure among female breakfast skippers, attributed to glucocorticoids and cortisol rhythm (76). As for adolescents, skipping breakfast may be a predictive factor for elevated blood pressure (77).

In addition, the absence of daily breakfast was associated with potential glycemic disorders, which resulted in elevated HbA1c and higher fasting plasma glucose from observational studies (78). Abnormal blood glucose rhythms and energy metabolism were also caused by this, including diurnal variation in blood glucose. In the potential sequence caused by skipping breakfast, glucose homeostasis may occur before energy balance (29, 79). Glucose status was improved if breakfast was added among those who habitually skip breakfast, including FBG and HOMA-IR (80, 81).

We sought to discover the effect of skipping breakfast on other cardiovascular risk factors (for example, LDL-C) with this meta-analysis, although the results were not statistically different. From an eating time perspective, breakfast skipping is similar to the strategy of delayed time-restricted feeding (dTRE). Compared to simply skipping breakfast, dTRE restricts feeding to at least after 10 a.m. until 8 a.m. the next day without energy restriction; in other words, this strategy skips breakfast (82). The latest meta-analysis indicated the benefits of dTRE on metabolic health since it increases the insulin sensitivity, lowers blood pressure, and reduces oxidative stress (83). Low-energy and high-energy dTRE reported different effects on lipid metabolism, glucose metabolism, and chronobiology, which involves hormone, gene, and protein expression levels (18). Considering that the included literature more closely resembles a regular diet that omits breakfast, maybe changes in energy are more likely to cause statistically significant changes in other cardiovascular risk factors (84–86). Due to the lack of direct evidence, this conjecture was derived indirectly.

In general, the contemporary dietary pattern is becoming increasingly diverse, emphasizing the need for vigilance regarding unhealthy eating behaviors or habits, such as skipping breakfast. Recent research has revealed a multitude of health outcomes and effects associated with breakfast consumption or omission. Furthermore, scholarly attention has been drawn to determining the healthier and more balanced breakfast options, highlighting the significance of considering aspects like the proportion of carbohydrates and proteins, as well as their respective sources (58, 87–92).

This current systematic review has several strengths and limitations. This is a comprehensive meta-analysis focusing on the effect of skipping breakfast on cardiovascular risk factors. For the first time, we noticed that gender differences, health status, and duration may affect the pooled results. Possible causes and mechanisms of pooled results were presented. Subject to the number of relevant literature and low to moderate level of evidence, more high-quality and large-sample studies are needed to verify or expand our findings.

## 5 Conclusion

Skipping breakfast may lead to weight loss and elevated levels of serum LDL-C. While it might be an alternative way to lose weight, it is a bad idea because of its negative effects on some cardiovascular risk factors.

## Author contributions

GS: Funding acquisition, Supervision, Validation, Writing – review & editing. JY: Conceptualization, Data curation, Formal Analysis, Methodology, Writing – original draft. JX: Conceptualization, Data curation, Writing – review & editing. DX: Investigation, Methodology, Software, Supervision, Writing – review & editing. YW: Formal Analysis, Project administration, Writing – review & editing. SY: Data curation, Formal Analysis, Resources, Investigation, Software, Writing – review & editing. YL: Project administration, Supervision, Validation, Conceptualization, Investigation, Writing – review & editing. HX: Visualization, Writing – review & editing. SW: Methodology, Project administration, Resources, Visualization, Writing – review & editing.
