# Supplementary material for: Effect of skipping breakfast on cardiovascular risk factors: a grade-assessed systematic review and meta-analysis of randomized controlled trials and prospective cohort studies
Source: Front Endocrinol (Lausanne). 2023 Nov 28;14:1256899. doi: 10.3389/fendo.2023.1256899 (PMC10715426; doi:10.3389/fendo.2023.1256899)
Supplement: Supplementary file 1 [file DataSheet_1.docx]

**Supplementary appendix**

**Supplement to:**

Effect of skipping breakfast on cardiovascular risk factors: a grade-assessed systematic review and meta-analysis of randomized controlled trials and prospective cohort studies

Junhui Yu, Jiayue Xia, Dengfeng Xu, Yuanyuan Wang, Shiyu Yin , Yifei Lu, Hui Xia, Shaokang Wang and Guiju Sun

Table of contents

Appendix 1: Risk of bias ............................................................................2

Appendix 2: Newcastle-Ottawa Scale (NOS)…………………………….3

Appendix 3: Grading of the evidence .........................................................4

Appendix 4: Funnel chart ………………………………………………...6

Appendix 5: Subgroup analysis………………………………………….13

Appendix 1: Risk of bias

| **Study** | **D1** | **D2** | **D3** | **D4** | **D5** | **Overall** |
| --- | --- | --- | --- | --- | --- | --- |
| Geliebter 2014 | 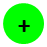 | 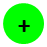 | 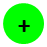 | 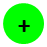 | 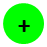 | 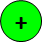 |
| Neumann 2016 | 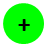 | 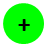 | 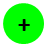 | 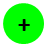 | 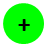 | 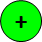 |
| Kobayashi 2013 | 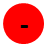 | 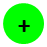 | 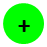 | 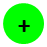 | 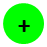 | 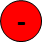 |
| Zhang 2017 | 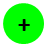 | 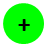 | 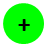 | 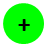 | 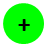 | 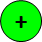 |
| Schlundt 1992 | 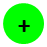 | 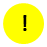 | 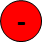 | 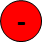 | 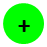 | 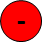 |
| Leidy 2015 | 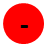 | 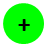 | 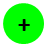 | 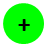 | 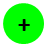 | 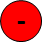 |
| LeCheminant 2016 | 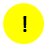 | 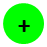 | 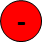 | 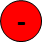 | 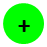 | 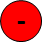 |
| Chowdhury 2016 | 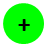 | 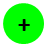 | 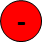 | 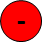 | 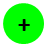 | 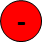 |
| Betts 2014 | 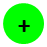 | 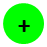 | 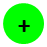 | 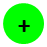 | 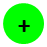 | 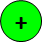 |
| Farshchi 2005 | 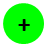 | 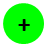 | 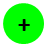 | 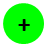 | 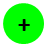 | 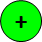 |

Appendix 2: Newcastle-Ottawa Scale (NOS)

| Study | **Representativeness of  the exposed cohort** | **Selection of the  nonexposed cohort** | **Ascertainment of  exposure** | **Demonstration that outcome of interest at start of study** | **Comparability of Cohorts on the basis of the  design or analysis** | **Assessment of  outcome** | **Follow-Up long enough for the outcome to occur** | **Adequacy of follow-up of cohorts** | **Grades** |
| --- | --- | --- | --- | --- | --- | --- | --- | --- | --- |
| Goff 2019 | ☆ | ☆ | ☆ | ☆ | ☆☆ | ☆ | ☆ | ☆ | 9 |

Appendix 3: Grading of the evidence

| **Quality assessment** | | | | | | | **No of patients** | | **Effect** | | **Quality** | **Importance** |  |
| --- | --- | --- | --- | --- | --- | --- | --- | --- | --- | --- | --- | --- | --- |
|  |  |  |  |  |  |  |  |  |  |  |  |  |  |
| **No of studies** | **Design** | **Risk of bias** | **Inconsistency** | **Indirectness** | **Imprecision** | **Other considerations** | **Skip breakfast** | **Breakfast** | **Relative (95% CI)** | **Absolute** |  |  |  |
| **Weight (follow-up median 90 days; measured with: Weight; Better indicated by lower values)** | | | | | | | | | | | | |  |
| 9 | randomized trials | no serious risk of bias | no serious inconsistency | serious^1^ | serious | none | 134 | 288 | - | MD 0.66 lower (1.09 to 0.23 lower) | ⊕⊕OO LOW | IMPORTANT |  |
| **BMI (follow-up median 42 days; Better indicated by lower values)** | | | | | | | | | | | | |  |
| 9 | randomized trials | no serious risk of bias | serious^2^ | no serious indirectness | serious^3^ | none | 844 | 827 | - | MD 0.13 lower (0.3 lower to 0.04 higher) | ⊕⊕OO LOW | IMPORTANT |  |
| **Waist (follow-up median 42 days; Better indicated by lower values)** | | | | | | | | | | | | |  |
| 5 | randomized trials | no serious risk of bias | serious^2^ | no serious indirectness | no serious imprecision | none | 72 | 178 | - | MD 0.374 lower (1.15 lower to 0.401 higher) | ⊕⊕⊕O MODERATE | IMPORTANT |  |
| **waist-to-hip ratio (follow-up median 36 days; Better indicated by lower values)** | | | | | | | | | | | | |  |
| 6 | randomized trials | no serious risk of bias | no serious inconsistency | no serious indirectness | no serious imprecision | none | 795 | 647 | - | MD 0.00 higher (0.01 lower to 0.01 higher) | ⊕⊕⊕⊕ HIGH | IMPORTANT |  |
| **SDP (follow-up median 29 weeks; Better indicated by lower values)** | | | | | | | | | | | | |  |
| 4 | randomized trials | no serious risk of bias | no serious inconsistency | no serious indirectness | serious^3^ | none | 777 | 727 | - | MD 0.972 higher (0 to 3.17 higher) | ⊕⊕⊕O MODERATE | IMPORTANT |  |
| **DBP (follow-up median 28 weeks; Better indicated by lower values)** | | | | | | | | | | | | |  |
| 2 | randomized trials | no serious risk of bias | no serious inconsistency | no serious indirectness | no serious imprecision | none | 33 | 141 | - | MD 1.17 lower (3.92 lower to 1.58 higher) | ⊕⊕⊕⊕ HIGH | IMPORTANT |  |
| **HDL-c (follow-up median 6 weeks; Better indicated by lower values)** | | | | | | | | | | | | |  |
| 7 | randomized trials | serious | no serious inconsistency | no serious indirectness^3^ | serious^3^ | none | 815 | 764 | - | MD 0.353 higher (0.53 lower to 1.24 higher) | ⊕⊕OO LOW | IMPORTANT |  |
| **LDL-c (follow-up median 4 weeks; Better indicated by lower values)** | | | | | | | | | | | | |  |
| 5 | randomized trials | serious | serious^2^ | no serious indirectness | no serious imprecision | none | 72 | 178 | - | MD 9.89 higher (5.14 to 14.63 higher) | ⊕⊕OO LOW | IMPORTANT |  |
| **TC (follow-up median 6 weeks; Better indicated by lower values)** | | | | | | | | | | | | |  |
| 7 | randomized trials | serious | serious^2^ | no serious indirectness | serious^3^ | none | 816 | 764 | - | MD 7.42 higher (2.74 lower to 17.58 higher) | ⊕OOO VERY LOW | IMPORTANT |  |
| **TG (follow-up median 6 weeks; Better indicated by lower values)** | | | | | | | | | | | | |  |
| 5 | randomized trials | no serious risk of bias | serious^2^ | no serious indirectness | no serious imprecision | none | 72 | 178 | - | MD 3.17 higher (3.55 lower to 9.89 higher) | ⊕⊕⊕O MODERATE | IMPORTANT |  |
| **FBG (follow-up median 5 weeks; Better indicated by lower values)** | | | | | | | | | | | | |  |
| 6 | randomized trials | no serious risk of bias | serious^2^ | no serious indirectness | no serious imprecision | none | 80 | 186 | - | MD 1.72 higher (5.53 lower to 8.98 higher) | ⊕⊕⊕O MODERATE | IMPORTANT |  |
| **HbA1c (follow-up median 10 years; Better indicated by lower values)** | | | | | | | | | | | | |  |
| 2 | randomized trials | no serious risk of bias | serious^2^ | no serious indirectness | serious^3^ | none | 744 | 586 | - | MD 0.05 higher (0.34 lower to 0.44 higher) | ⊕⊕OO LOW | IMPORTANT |  |
| **HOMA-IR (follow-up median 29 weeks; Better indicated by lower values)** | | | | | | | | | | | | |  |
| 4 | randomized trials | no serious risk of bias | serious^2^ | no serious indirectness | serious^3^ | none | 124 | 168 | - | MD 0.15 lower (0.66 lower to 0.36 higher) | ⊕⊕OO LOW | IMPORTANT |  |


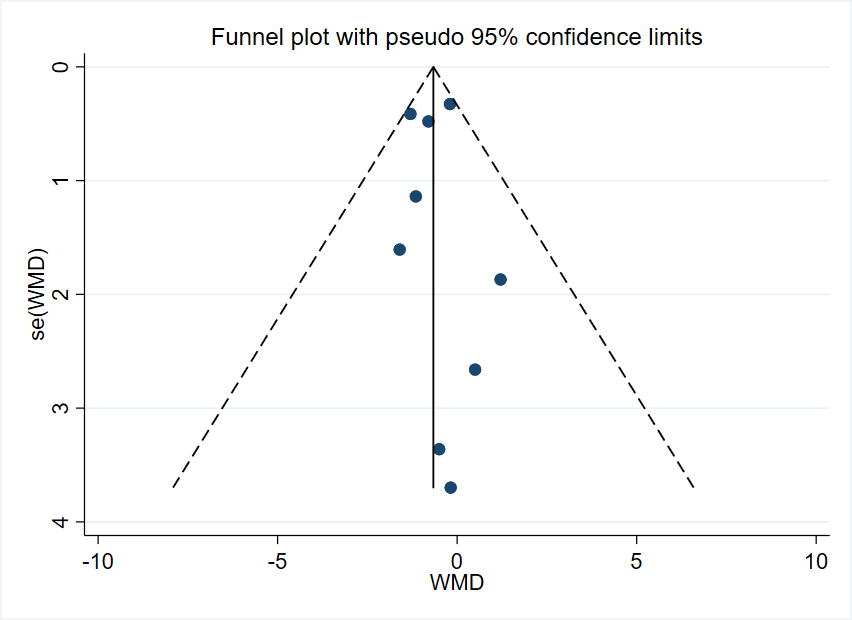
Appendix 4: Funnel chart


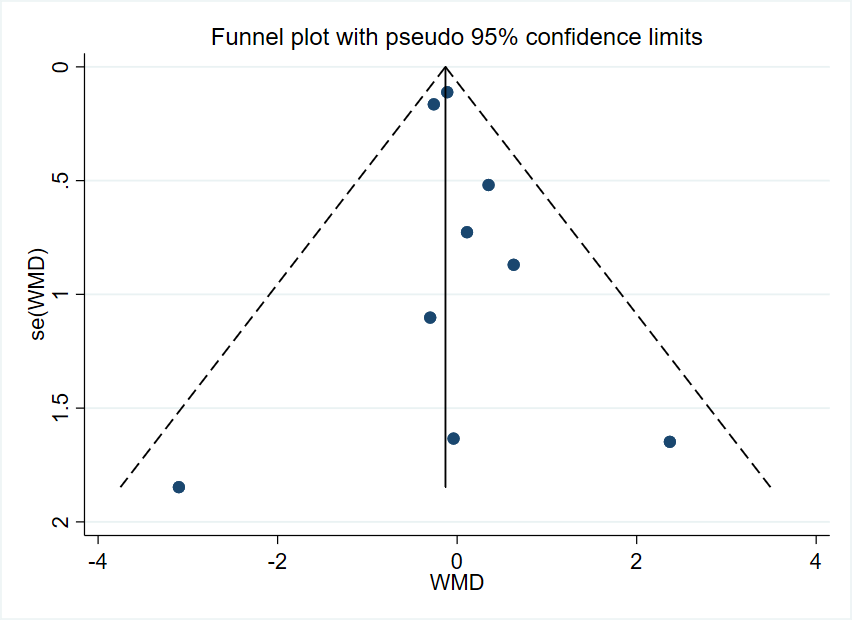
Funnel plots for exhibition of publication bias for weight.

Funnel plots for exhibition of publication bias for BMI.


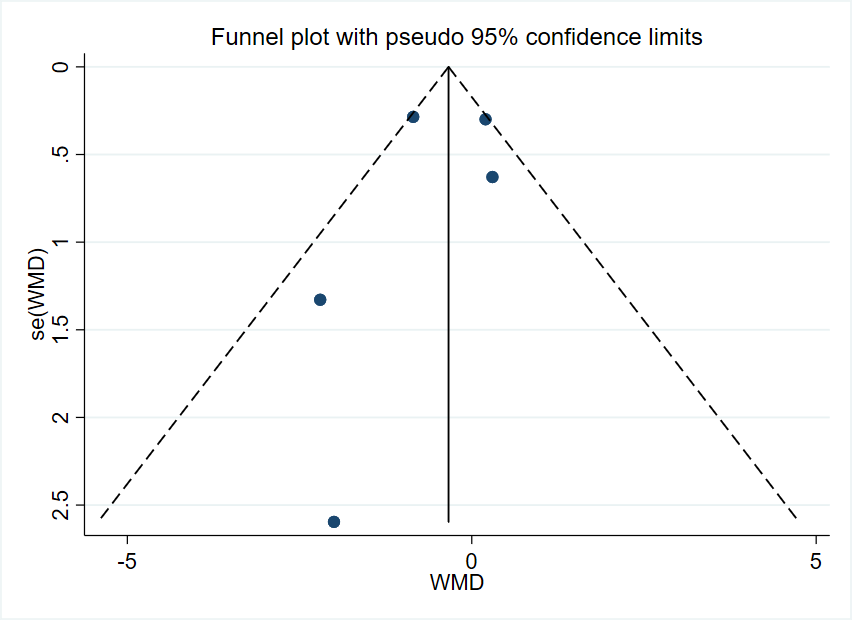


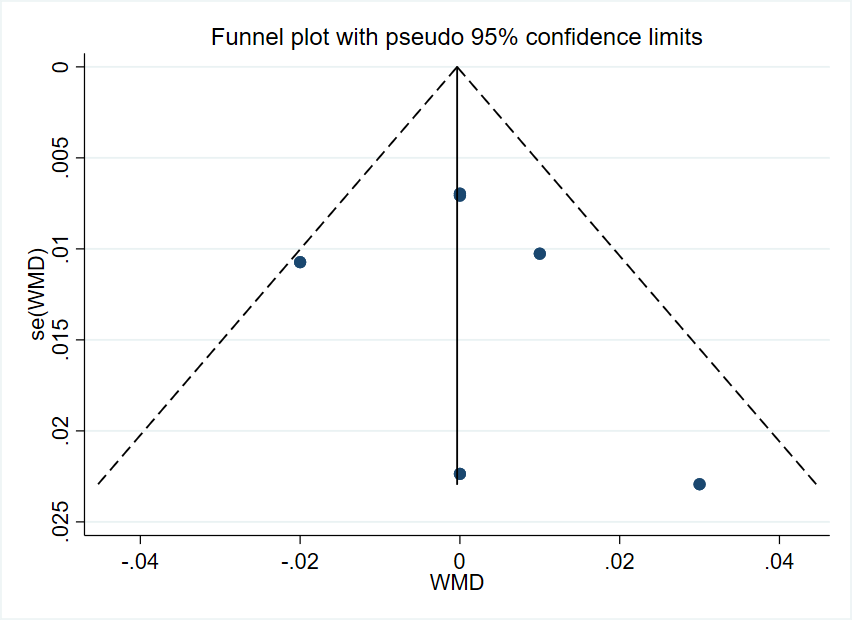
Funnel plots for exhibition of publication bias for waist.

Funnel plots for exhibition of publication bias for waist to hip ratio.


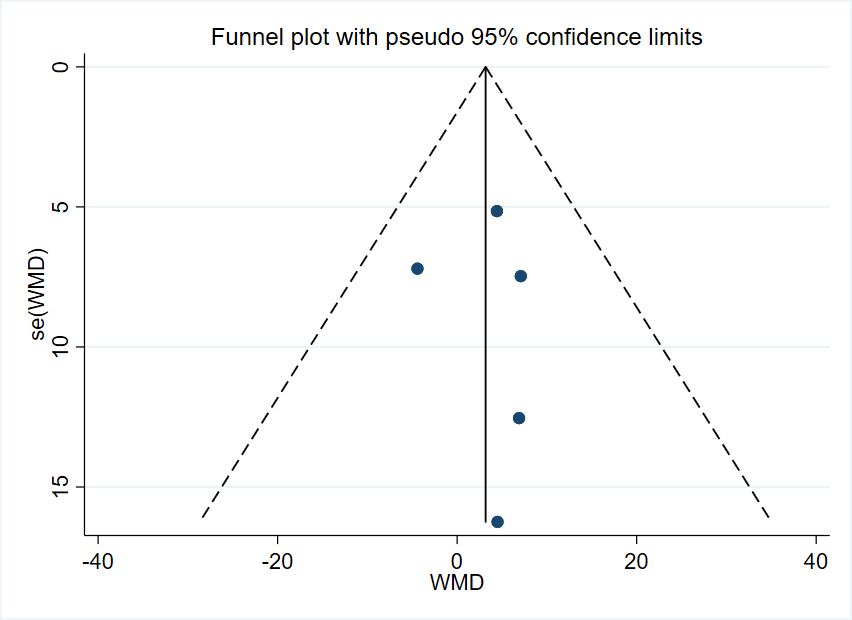

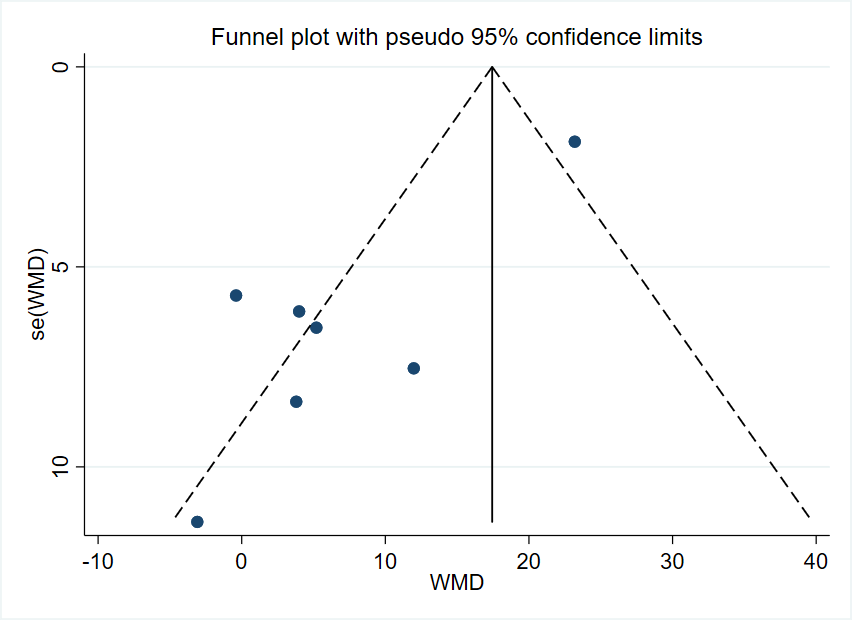
Funnel plots for exhibition of publication bias for TC.

Funnel plots for exhibition of publication bias for TG.


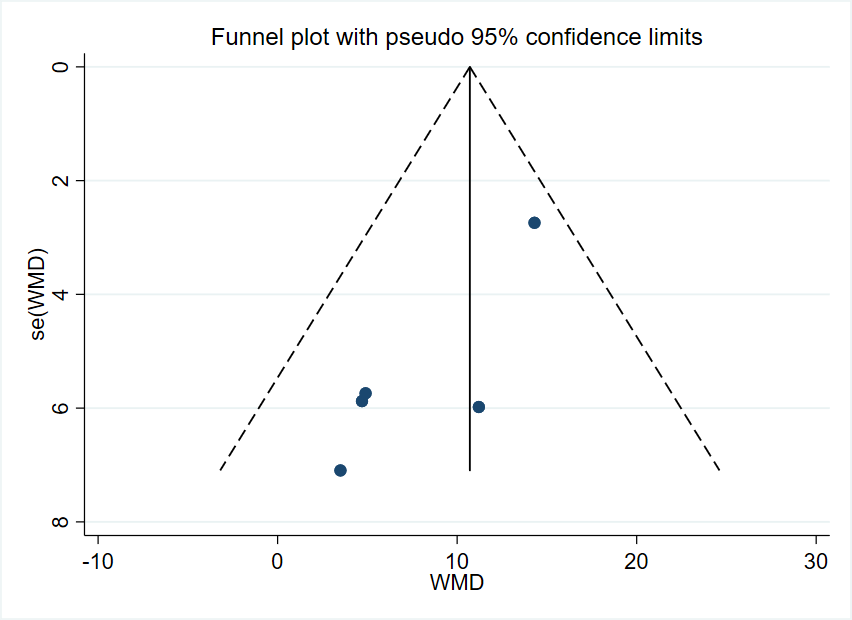

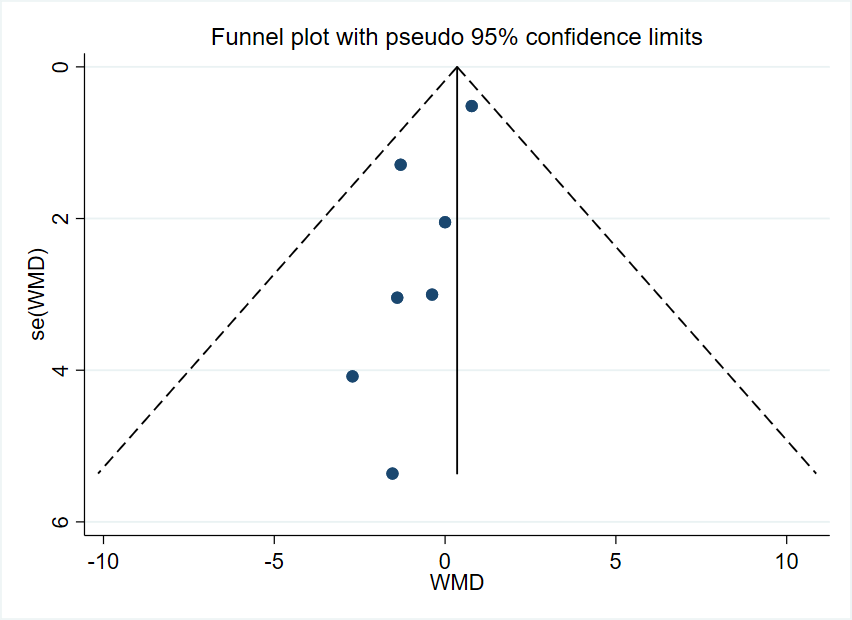
Funnel plots for exhibition of publication bias for HDL-C.

Funnel plots for exhibition of publication bias for LDL-C.


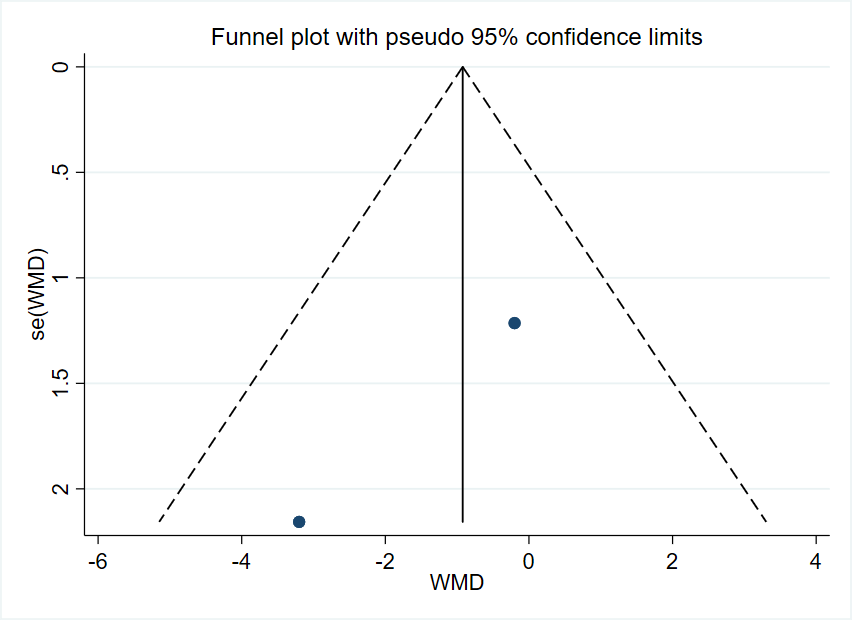

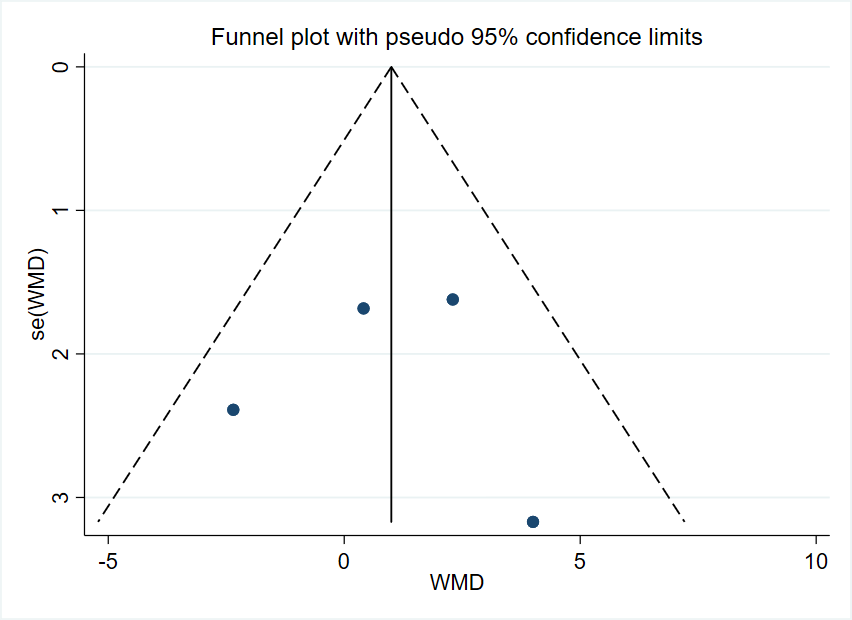
Funnel plots for exhibition of publication bias for SBP.

Funnel plots for exhibition of publication bias for DBP.


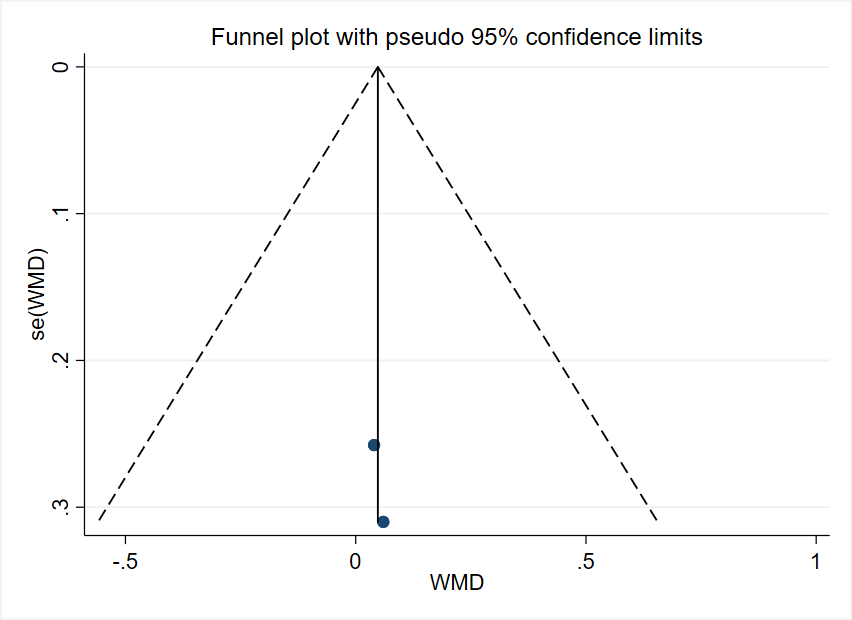

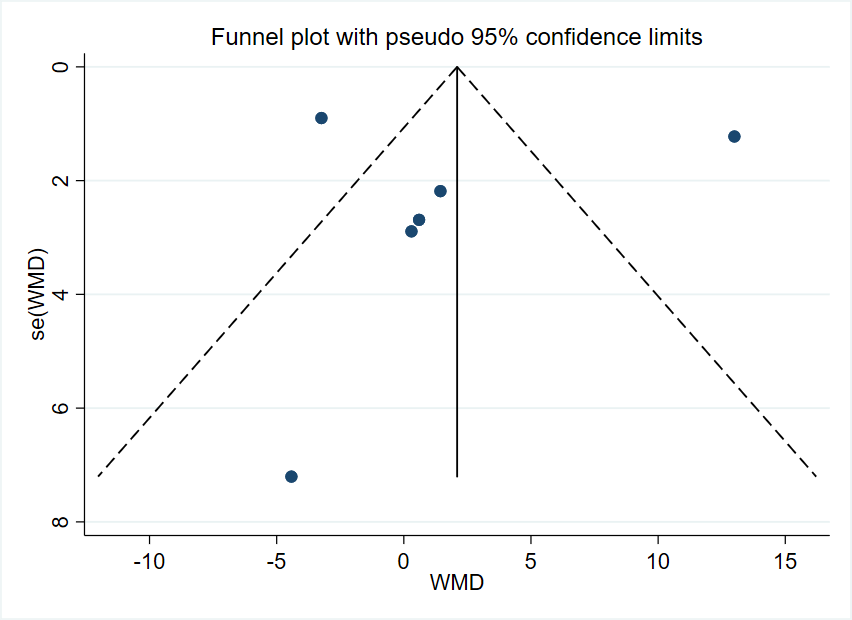
Funnel plots for exhibition of publication bias for FBG.

Funnel plots for exhibition of publication bias for HbA1c.


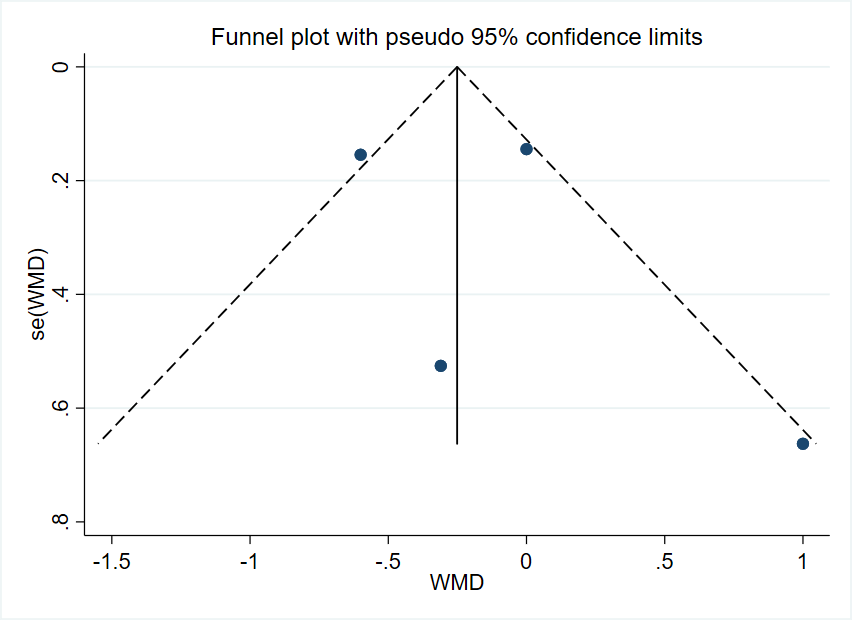
Funnel plots for exhibition of publication bias for HOMA-IR.

Appendix 5: Subgroup analysis

| **Subgroup** | | **Weight** | | | | | |  |
| --- | --- | --- | --- | --- | --- | --- | --- | --- |
|  |  |  |  |  |  |  |  |  |
|  |  | **No. of Trials** | **WMD** | | ***P*** | **I^2^(%)** | ***P* value of heterogeneity** |  |
|  |  |  | **mean** | **95%CI** |  |  |  |  |
| **Overall** | | 9 | ﹣0.66 | ﹣1.09, ﹣0.24 | 0.002 | 0.0 | 0.626 |  |
| **Physiological condition** | |  |  |  |  |  |  |  |
| healthy state | | 5 | ﹣0.64 | ﹣1.26, ﹣0.03 | 0.410 | 11.9 | 0.338 |  |
| disease state | | 4 | ﹣0.80 | ﹣1.62, ﹣0.24 | 0.054 | 0.0 | 0.626 |  |
| **Duration** | |  |  |  |  |  |  |  |
| ≤4 weeks | | 4 | ﹣1.23 | ﹣2.02, ﹣0.44 | 0.002 | 0.0 | 0.901 |  |
| ＞4 weeks | | 5 | ﹣0.43 | ﹣0.93, 0.07 | 0.094 | 0.0 | 0.594 |  |
| **Gender** | |  |  |  |  |  |  |  |
| male | | ﹣ | ﹣ | ﹣ | ﹣ | ﹣ | ﹣ |  |
| female | | 4 | ﹣0.81 | ﹣2.7, 1.08 | 0.399 | 0.0 | 0.947 |  |
| mixed |  | 5 | ﹣0.70 | ﹣1.29, ﹣0.10 | 0.022 | 31.0 | 0.215 |  |
| **Type of breakfast** | |  |  |  |  |  |  |  |
| normal breakfast | | 6 | ﹣0.70 | ﹣1.13, ﹣0.27 | 0 | 0.0 | 0.421 |  |
| fortified breakfast | | 3 | 0.81 | ﹣1.96, 3.58 | 0.57 | 0.0 | 0.937 |  |
| **Study design** | |  |  |  |  |  |  |  |
| randomized controlled trial | | 8 | ﹣0.67 | ﹣1.10, ﹣0.24 | 0.002 | 0.0 | 0.540 |  |
| cross‐over trial | | 1 | 0.50 | ﹣4.72, 5.72 | 0.851 | 56.2 | 0.025 |  |
| prospective cohort study | | ﹣ | ﹣ | ﹣ | ﹣ | ﹣ | ﹣ |  |

| **Subgroup** | | **BMI** | | | | | |  |
| --- | --- | --- | --- | --- | --- | --- | --- | --- |
|  |  |  |  |  |  |  |  |  |
|  |  | **No. of Trials** | **WMD** | | ***P*** | **I^2^(%)** | ***P* value of heterogeneity** |  |
|  |  |  | **mean** | **95%CI** |  |  |  |  |
| **Overall** | | 9 | ﹣0.13 | ﹣0.30, 0.04 | 0.144 | 0.0 | 0.505 |  |
| **Physiological condition** | |  |  |  |  |  |  |  |
| healthy state | | 6 | ﹣0.08 | ﹣0.30, 0.13 | 0.435 | 0.0 | 0.691 |  |
| disease state | | 3 | ﹣0.19 | ﹣0.98, 0.60 | 0.641 | 45.8 | 0.158 |  |
| **Duration** | |  |  |  |  |  |  |  |
| ≤4 weeks | | 3 | ﹣0.02 | ﹣1.13, 1.10 | 0.976 | 0.0 | 0.953 |  |
| ＞4 weeks | | 6 | ﹣0.11 | ﹣0.41, 0.20 | 0.490 | 30.1 | 0.209 |  |
| **Gender** | |  |  |  |  |  |  |  |
| male | | ﹣ | ﹣ | ﹣ | ﹣ | ﹣ | ﹣ |  |
| female | | 3 | ﹣0.02 | ﹣1.12, 1.10 | 0.976 | 0.0 | 0.953 |  |
| mixed |  | 6 | ﹣0.11 | ﹣0.41, 0.20 | 0.490 | 30.1 | 0.209 |  |
| **Type of breakfast** | |  |  |  |  |  |  |  |
| normal breakfast | | 6 | ﹣0.15 | ﹣0.43, 0.12 | 0.28 | 20.4 | 0.279 |  |
| fortified breakfast | | 3 | 0.25 | ﹣0.55, 1.05 | 0.54 | 0.0 | 0.949 |  |
| **Study design** | |  |  |  |  |  |  |  |
| randomized controlled trial | | 6 | ﹣0.15 | ﹣0.33, 0.03 | 0.098 | 78.3 | 0.000 |  |
| cross‐over trial | | 1 | 0.11 | ﹣1.31, 1.53 | 0.880 | ﹣ | ﹣ |  |
| prospective cohort study | | 2 | 1.01 | ﹣0.50, 2.52 | 0.190 | 0.0 | 0.350 |  |

| **Subgroup** | | **Waist circumference** | | | | | |  |
| --- | --- | --- | --- | --- | --- | --- | --- | --- |
|  |  |  |  |  |  |  |  |  |
|  |  | **No. of Trials** | **WMD** | | ***P*** | **I^2^(%)** | ***P* value of heterogeneity** |  |
|  |  |  | **mean** | **95%CI** |  |  |  |  |
| **Overall** | | 5 | ﹣0.37 | ﹣1.15, 0.40 | 0.344 | 59.4 | 0.043 |  |
| **Physiological condition** | |  |  |  |  |  |  |  |
| healthy state | | 3 | ﹣0.51 | ﹣1.40, 0.37 | 0.258 | 34.3 | 0.218 |  |
| disease state | | 2 | ﹣0.06 | ﹣0.07, ﹣0.05 | 0.571 | 67.8 | 0.078 |  |
| **Duration** | |  |  |  |  |  |  |  |
| ≤4 weeks | | 2 | ﹣0.86 | ﹣1.42, ﹣0.31 | 0.002 | 0.0 | 0.660 |  |
| ＞4 weeks | | 3 | 0.00 | ﹣0.87, 0.87 | 0.995 | 37.6 | 0.202 |  |
| **Gender** | |  |  |  |  |  |  |  |
| male | | ﹣ | ﹣ | ﹣ | ﹣ | ﹣ | ﹣ |  |
| female | | 1 | ﹣2.00 | ﹣7.09, 3.09 | 0.441 | ﹣ | ﹣ |  |
| mixed |  | 4 | ﹣0.34 | ﹣1.16, 0.47 | 0.410 | 68.3 | 0.024 |  |
| **Type of breakfast** | |  |  |  |  |  |  |  |
| normal breakfast | | 4 | ﹣0.34 | ﹣1.16, 0.47 | 0.410 | 68.3 | 0.024 |  |
| fortified breakfast | | 1 | ﹣2.00 | ﹣7.09, 3.09 | 0.441 | ﹣ | ﹣ |  |
| **Study design** | |  |  |  |  |  |  |  |
| randomized controlled trial | | 4 | ﹣0.34 | ﹣1.16, 0.47 | 0.410 | 68.3 | 0.024 |  |
| cross‐over trial | | 1 | ﹣2.00 | ﹣7.09, 3.09 | 0.441 | ﹣ | ﹣ |  |
| prospective cohort study | | ﹣ | ﹣ | ﹣ | ﹣ | ﹣ | ﹣ |  |

| **Subgroup** | | **Waist﹣to﹣hip ratio** | | | | | |  |
| --- | --- | --- | --- | --- | --- | --- | --- | --- |
|  |  |  |  |  |  |  |  |  |
|  |  | **No. of Trials** | **WMD** | | ***P*** | **I^2^(%)** | ***P* value of heterogeneity** |  |
|  |  |  | **mean** | **95%CI** |  |  |  |  |
| **Overall** | | 6 | 0.00 | ﹣0.01, 0.01 | 0.956 | 18.4 | 0.294 |  |
| **Physiological condition** | |  |  |  |  |  |  |  |
| healthy state | | 5 | 0.00 | ﹣0.01, 0.01 | 0.513 | 0.0 | 0.693 |  |
| disease state | | 1 | ﹣0.02 | ﹣0.04, 0.00 | 0.062 | ﹣ | ﹣ |  |
| **Duration** | |  |  |  |  |  |  |  |
| ≤4 weeks | | 2 | 0.00 | ﹣0.01, 0.01 | 1.000 | 0.0 | 1.000 |  |
| ＞4 weeks | | 4 | 0.00 | ﹣0.02, 0.02 | 0.964 | 51.0 | 0.106 |  |
| **Gender** | |  |  |  |  |  |  |  |
| male | | ﹣ | ﹣ | ﹣ | ﹣ | ﹣ | ﹣ |  |
| female | | 1 | 0.00 | ﹣0.04, 0.04 | 0.978 | ﹣ | ﹣ |  |
| mixed |  | 5 | 0.00 | ﹣0.01, 0.01 | 0.956 | 34.7 | 0.190 |  |
| **Type of breakfast** | |  |  |  |  |  |  |  |
| normal breakfast | | 5 | 0.00 | ﹣0.01, 0.01 | 0.978 | 34.7 | 0.190 |  |
| fortified breakfast | | 1 | 0.00 | ﹣0.04, 0.04 | 1.000 | ﹣ | ﹣ |  |
| **Study design** | |  |  |  |  |  |  |  |
| randomized controlled trial | | 3 | 0.00 | ﹣0.02, 0.02 | 0.441 | 30.1 | 0.239 |  |
| cross‐over trial | | 1 | 0.00 | ﹣0.04, 0.04 | 1.000 | ﹣ | ﹣ |  |
| prospective cohort study | | 2 | 0.01 | ﹣0.01, 0.03 | 0.155 | 0.0 | 0.426 |  |

| **Subgroup** | | **TC** | | | | | |  |
| --- | --- | --- | --- | --- | --- | --- | --- | --- |
|  |  |  |  |  |  |  |  |  |
|  |  | **No. of Trials** | **WMD** | | ***P*** | **I^2^(%)** | ***P* value of heterogeneity** |  |
|  |  |  | **mean** | **95%CI** |  |  |  |  |
| **Overall** | | 7 | 7.42 | ﹣2.74, 17.58 | 0.152 | 41.0 | 0.045 |  |
| **Physiological condition** | |  |  |  |  |  |  |  |
| healthy state | | 5 | 8.24 | ﹣4.52, 21.37 | 0.202 | 83.6 | 0.000 |  |
| disease state | | 2 | 4.56 | ﹣4.18, 13.30 | 0.306 | 0.0 | 0.893 |  |
| **Duration** | |  |  |  |  |  |  |  |
| ≤4 weeks | | 2 | 19.96 | 10.01, 29.91 | 0.000 | 52.0 | 0.149 |  |
| ＞4 weeks | | 5 | 2.41 | ﹣3.70, 8.52 | 0.440 | 0.0 | 0.945 |  |
| **Gender** | |  |  |  |  |  |  |  |
| male | | ﹣ | ﹣ | ﹣ | ﹣ | ﹣ | ﹣ |  |
| female | | 1 | 11.98 | ﹣2.79, 26.75 | 0.112 | ﹣ | ﹣ |  |
| mixed |  | 6 | 6.55 | ﹣5.08, 18.18 | 0.270 | 85.0 | 0.000 |  |
| **Type of breakfast** | |  |  |  |  |  |  |  |
| normal breakfast | | 6 | 6.55 | ﹣5.08, 18.18 | 0.270 | 85.0 | 0.000 |  |
| fortified breakfast | | 1 | 11.98 | ﹣2.79, 26.75 | 0.112 | ﹣ | ﹣ |  |
| **Study design** | |  |  |  |  |  |  |  |
| randomized controlled trial | | 4 | 10.11 | ﹣2.79, 23.01 | 0.125 | 83.9 | 0.000 |  |
| cross‐over trial | | 1 | ﹣0.26 | ﹣0.34, ﹣0.18 | 0.112 | ﹣ | ﹣ |  |
| prospective cohort study | | 2 | ﹣0.93 | ﹣10.94, 9.08 | 0.855 | 0.0 | 0.832 |  |

| **Subgroup** | | **TG** | | | | | |  |
| --- | --- | --- | --- | --- | --- | --- | --- | --- |
|  |  |  |  |  |  |  |  |  |
|  |  | **No. of Trials** | **WMD** | | ***P*** | **I^2^(%)** | ***P* value of heterogeneity** |  |
|  |  |  | **mean** | **95%CI** |  |  |  |  |
| **Overall** | | 5 | 3.17 | ﹣3.55, 9.89 | 0.355 | 0.0 | 0.819 |  |
| **Physiological condition** | |  |  |  |  |  |  |  |
| healthy state | | 3 | 2.79 | ﹣4.37, 9.95 | 0.445 | 0.0 | 0.488 |  |
| disease state | | 2 | 6.00 | ﹣13.45, 25.46 | 0.545 | 0.0 | 0.907 |  |
| **Duration** | |  |  |  |  |  |  |  |
| ≤4 weeks | | 2 | 5.29 | ﹣3.02, 13.60 | 0.212 | 0.0 | 0.769 |  |
| ＞4 weeks | | 3 | ﹣0.82 | ﹣12.25, 10.60 | 0.888 | 0.0 | 0.692 |  |
| **Gender** | |  |  |  |  |  |  |  |
| male | | ﹣ | ﹣ | ﹣ | ﹣ | ﹣ | ﹣ |  |
| female | | 1 | 7.09 | ﹣7.55, 21.73 | 0.343 | ﹣ | ﹣ |  |
| mixed |  | 4 | 2.13 | ﹣5.44, 9.69 | 0.582 | 0.0 | 0.755 |  |
| **Type of breakfast** | |  |  |  |  |  |  |  |
| normal breakfast | | 3 | ﹣0.82 | ﹣12.25, 10.60 | 0.89 | 0.0 | 0.692 |  |
| fortified breakfast | | 2 | 5.29 | ﹣3.02, 13.60 | 0.212 | 0.0 | 0.769 |  |
| **Study design** | |  |  |  |  |  |  |  |
| randomized controlled trial | | 4 | 2.13 | ﹣5.44, 9.69 | 0.582 | 0.0 | 0.755 |  |
| cross‐over trial | | 1 | 7.09 | ﹣7.55, 21.73 | 0.343 | ﹣ | ﹣ |  |
| prospective cohort study | | ﹣ | ﹣ | ﹣ | ﹣ | ﹣ | ﹣ |  |

| **Subgroup** | | **HDL﹣C** | | | | | |  |
| --- | --- | --- | --- | --- | --- | --- | --- | --- |
|  |  |  |  |  |  |  |  |  |
|  |  | **No. of Trials** | **WMD** | | ***P*** | **I^2^(%)** | ***P* value of heterogeneity** |  |
|  |  |  | **mean** | **95%CI** |  |  |  |  |
| **Overall** | | 7 | 0.35 | ﹣0.53, 1.24 | 0.435 | 0.0 | 0.753 |  |
| **Physiological condition** | |  |  |  |  |  |  |  |
| healthy state | | 5 | 0.64 | ﹣0.32, 1.59 | 0.194 | 0.0 | 0.890 |  |
| disease state | | 2 | ﹣1.32 | ﹣3.64, 1.01 | 0.268 | 0.0 | 0.976 |  |
| **Duration** | |  |  |  |  |  |  |  |
| ≤4 weeks | | 2 | 0.76 | ﹣0.25, 1.77 | 0.140 | 0.0 | 0.667 |  |
| ＞4 weeks | | 5 | ﹣1.02 | ﹣2.87, 0.84 | 0.282 | 0.0 | 0.971 |  |
| **Gender** | |  |  |  |  |  |  |  |
| male | | ﹣ | ﹣ | ﹣ | ﹣ | ﹣ | ﹣ |  |
| female | | 1 | ﹣1.54 | ﹣12.05, 8.97 | 0.774 | ﹣ | ﹣ |  |
| mixed |  | 6 | 0.37 | ﹣0.52, 1.26 | 0.419 | 0.0 | 0.654 |  |
| **Type of breakfast** | |  |  |  |  |  |  |  |
| normal breakfast | | 6 | 0.37 | ﹣0.52, 1.26 | 0.419 | 0.0 | 0.654 |  |
| fortified breakfast | | 1 | ﹣1.54 | ﹣12.05, 8.97 | 0.774 | ﹣ | ﹣ |  |
| **Study design** | |  |  |  |  |  |  |  |
| randomized controlled trial | | 4 | 0.42 | ﹣0.48, 1.33 | 0.359 | 0.0 | 0.447 |  |
| cross‐over trial | | 1 | ﹣1.54 | ﹣12.05, 8.97 | 0.774 | ﹣ | ﹣ |  |
| prospective cohort study | | 2 | ﹣1.20 | ﹣5.94, 3.54 | 0.620 | 0.0 | 0.646 |  |

| **Subgroup** | | **LDL﹣C** | | | | | |  |
| --- | --- | --- | --- | --- | --- | --- | --- | --- |
|  |  |  |  |  |  |  |  |  |
|  |  | **No. of Trials** | **WMD** | | ***P*** | **I^2^(%)** | ***P* value of heterogeneity** |  |
|  |  |  | **mean** | **95%CI** |  |  |  |  |
| **Overall** | | 5 | 9.89 | 5.14, 14.63 | 0.000 | 17.3 | 0.305 |  |
| **Physiological condition** | |  |  |  |  |  |  |  |
| healthy state | | 3 | 12.50 | 7.63, 17.73 | 0.000 | 4.2 | 0.352 |  |
| disease state | | 2 | 4.80 | ﹣3.24, 12.85 | 0.242 | 0.0 | 0.981 |  |
| **Duration** | |  |  |  |  |  |  |  |
| ≤4 weeks | | 2 | 13.77 | 8.89, 18.66 | 0.000 | 0.0 | 0.637 |  |
| ＞4 weeks | | 3 | 4.48 | ﹣2.49, 11.44 | 0.208 | 0.0 | 0.987 |  |
| **Gender** | |  |  |  |  |  |  |  |
| male | | ﹣ | ﹣ | ﹣ | ﹣ | ﹣ | ﹣ |  |
| female | | 1 | 11.21 | ﹣0.51, 22.93 | 0.061 | ﹣ | ﹣ |  |
| mixed |  | 5 | 8.77 | 2.56, 14.98 | 0.006 | 37.9 | 0.185 |  |
| **Type of breakfast** | |  |  |  |  |  |  |  |
| normal breakfast | | 3 | 4.48 | ﹣2.49, 11.44 | 0.208 | 0.0 | 0.987 |  |
| fortified breakfast | | 2 | 13.77 | 8.89, 18.66 | 0.000 | 0.0 | 0.637 |  |
| **Study design** | |  |  |  |  |  |  |  |
| randomized controlled trial | | 4 | 8.77 | 2.56, 14.98 | 0.006 | 37.9 | 0.185 |  |
| cross‐over trial | | 1 | 11.21 | ﹣0.51, 22.93 | 0.061 | ﹣ | ﹣ |  |
| prospective cohort study | | ﹣ | ﹣ | ﹣ | ﹣ | ﹣ | ﹣ |  |

| **Subgroup** | | **SBP** | | | | | |  |
| --- | --- | --- | --- | --- | --- | --- | --- | --- |
|  |  |  |  |  |  |  |  |  |
|  |  | **No. of Trials** | **WMD** | | ***P*** | **I^2^(%)** | ***P* value of heterogeneity** |  |
|  |  |  | **mean** | **95%CI** |  |  |  |  |
| **Overall** | | 4 | 0.97 | ﹣1.23, 3.17 | 0.386 | 17.3 | 0.305 |  |
| **Physiological condition** | |  |  |  |  |  |  |  |
| healthy state | | 3 | 0.26 | ﹣2.68, 3.19 | 0.156 | 22.8 | 0.274 |  |
| disease state | | 1 | 2.30 | ﹣0.88, 5.48 | 0.386 | ﹣ | ﹣ |  |
| **Duration** | |  |  |  |  |  |  |  |
| ≤4 weeks | | 1 | ﹣2.35 | ﹣7.03, 2.33 | 0.325 | ﹣ | ﹣ |  |
| ＞4 weeks | | 3 | 1.70 | ﹣0.44, 3.85 | 0.120 | 0.0 | 0.535 |  |
| **Gender** | |  |  |  |  |  |  |  |
| male | | ﹣ | ﹣ | ﹣ | ﹣ | ﹣ | ﹣ |  |
| female | | ﹣ | ﹣ | ﹣ | ﹣ | ﹣ | ﹣ |  |
| mixed |  | 4 | 0.97 | ﹣1.23, 3.17 | 0.864 | 17.3 | 0.305 |  |
| **Type of breakfast** | |  |  |  |  |  |  |  |
| normal breakfast | | 4 | 0.97 | ﹣1.23, 3.17 | 0.864 | 17.3 | 0.305 |  |
| fortified breakfast | | ﹣ | ﹣ | ﹣ | ﹣ | ﹣ | ﹣ |  |
| **Study design** | |  |  |  |  |  |  |  |
| randomized controlled trial | | 2 | 0.31 | ﹣4.20, 4.82 | 0.894 | 61.5 | 0.107 |  |
| cross‐over trial | | ﹣ | ﹣ | ﹣ | ﹣ | ﹣ | ﹣ |  |
| prospective cohort study | | 2 | 1.20 | ﹣1.71, 4.11 | 0.420 | 0.0 | 0.317 |  |

| **Subgroup** | | **FBG** | | | | | |  |
| --- | --- | --- | --- | --- | --- | --- | --- | --- |
|  |  |  |  |  |  |  |  |  |
|  |  | **No. of Trials** | **WMD** | | ***P*** | **I^2^(%)** | ***P* value of heterogeneity** |  |
|  |  |  | **mean** | **95%CI** |  |  |  |  |
| **Overall** | | 6 | 0.00 | ﹣0.05, 0.05 | 0.642 | 97.2 | 0.000 |  |
| **Physiological condition** | |  |  |  |  |  |  |  |
| healthy state | | 4 | 2.27 | ﹣7.82, 12.36 | 0.660 | 97.4 | 0.000 |  |
| disease state | | 2 | 0.46 | ﹣3.40, 4.32 | 0.815 | 0.0 | 0.939 |  |
| **Duration** | |  |  |  |  |  |  |  |
| ≤4 weeks | | 3 | 3.74 | ﹣7.54, 15.03 | 0.516 | 98.3 | 0.000 |  |
| ＞4 weeks | | 3 | 0.12 | ﹣3.60, 3.85 | 0.949 | 0.0 | 0.805 |  |
| **Gender** | |  |  |  |  |  |  |  |
| male | | 1 | 13.00 | 10.60, 15.40 | 0.000 | ﹣ | ﹣ |  |
| female | | 1 | 1.44 | ﹣2.84, 5.72 | 0.510 | ﹣ | ﹣ |  |
| mixed |  | 4 | ﹣2.62 | ﹣4.22, ﹣1.03 | 0.001 | 0.0 | 0.393 |  |
| **Type of breakfast** | |  |  |  |  |  |  |  |
| normal breakfast | | 3 | 0.12 | ﹣3.60, 3.85 | 0.95 | 0.0 | 0.805 |  |
| fortified breakfast | | 3 | 3.74 | ﹣7.54, 15.03 | 0.52 | 98.3 | 0.000 |  |
| **Study design** | |  |  |  |  |  |  |  |
| randomized controlled trial | | 4 | ﹣2.62 | ﹣4.22, ﹣1.03 | 0.001 | 0.0 | 0.393 |  |
| cross‐over trial | | 2 | 7.36 | ﹣3.96, 18.69 | 0.203 | 95.3 | 0.000 |  |
| prospective cohort study | | ﹣ | ﹣ | ﹣ | ﹣ | ﹣ | ﹣ |  |

| **Subgroup** | | **HOMA﹣IR** | | | | | |  |
| --- | --- | --- | --- | --- | --- | --- | --- | --- |
|  |  |  |  |  |  |  |  |  |
|  |  | **No. of Trials** | **WMD** | | ***P*** | **I^2^(%)** | ***P* value of heterogeneity** |  |
|  |  |  | **mean** | **95%CI** |  |  |  |  |
| **Overall** | | 4 | ﹣0.15 | ﹣0.66, 0.36 | 0.558 | 74.3 | 0.009 |  |
| **Physiological condition** | |  |  |  |  |  |  |  |
| healthy state | | 2 | ﹣0.30 | ﹣0.89, 0.29 | 0.321 | 87.6 | 0.005 |  |
| disease state | | 2 | 0.28 | ﹣1.00, 1,56 | 0.664 | 58.3 | 0.121 |  |
| **Duration** | |  |  |  |  |  |  |  |
| ≤4 weeks | | 1 | ﹣0.60 | ﹣0.90, ﹣0.30 | 0.000 | ﹣ | ﹣ |  |
| ＞4 weeks | | 3 | 0.06 | ﹣0.40, 0.52 | 0.798 | 23.1 | 0.272 |  |
| **Gender** | |  |  |  |  |  |  |  |
| male | | ﹣ | ﹣ | ﹣ | ﹣ | ﹣ | ﹣ |  |
| female | | ﹣ | ﹣ | ﹣ | ﹣ | ﹣ | ﹣ |  |
| mixed |  | 4 | ﹣0.15 | ﹣0.66, 0.36 | 0.558 | 74.3 | 0.009 |  |
| **Type of breakfast** | |  |  |  |  |  |  |  |
| normal breakfast | | 4 | ﹣0.15 | ﹣0.66, 0.36 | 0.558 | 74.3 | 0.009 |  |
| fortified breakfast | | ﹣ | ﹣ | ﹣ | ﹣ | ﹣ | ﹣ |  |
| **Study design** | |  |  |  |  |  |  |  |
| randomized controlled trial | | 4 | ﹣0.15 | ﹣0.66, 0.36 | 0.558 | 74.3 | 0.009 |  |
| cross‐over trial | | ﹣ | ﹣ | ﹣ | ﹣ | ﹣ | ﹣ |  |
| prospective cohort study | | ﹣ | ﹣ | ﹣ | ﹣ | ﹣ | ﹣ |  |
